# Evaluation of a Simplified Loops System for Emergency Rescue Lifting of the Stranded or Recumbent Horse

**DOI:** 10.3390/ani9080511

**Published:** 2019-07-31

**Authors:** John Madigan, Lais Costa, Samantha Nieves, Molly Horgan, Kirsten Weberg, Monica Aleman

**Affiliations:** Department of Medicine and Epidemiology, School of Veterinary Medicine, University of California, Davis, CA 95616, USA

**Keywords:** equine, sling lifting, rescue

## Abstract

**Simple Summary:**

Horses, once stranded or recumbent, can hurt themselves and endanger the personnel trying to rescue them. Successful rescuing of these horses often requires emergency lifting. The objective of this study is to describe a new sling system for short-term lifting of horses. This simple system, called here the Loop Vertical Lift System, or Loops System, utilizes commercially available, and reasonably priced equipment that when used correctly may save lives of stranded horses needing emergency rescue and short-term lifting.

**Abstract:**

Stranded and recumbent equids often require emergency rescue, and a successful rescue often requires vertical lifting of the animal. Currently, the devices used for vertical lifting of equids are not readily available at an incident or urgent situation. The current study describes and evaluates the use of a simple lift device utilizing commercially available, and reasonably priced, equipment. The system, referred to as the Loop Vertical Lift System or Loops System, is basically composed of four round slings placed in such a way that utilizes the skeletal system for support. The study demonstrates the lifting of six standing, sedated adult horses for 3 min without adverse effects. In conclusion, this novel lift system is an affordable, practical and quick alternative to rescue a stranded or recumbent horse that requires a brief vertical lift of the animal. In contrast, for longer-term lifting and support, other devices such as the UC Davis Large Animal Lift, the Anderson Sling Support Device, or the Animal Rescue and Transport Sling (ARTS) should be used as deemed appropriate.

## 1. Introduction

Horses can become trapped in confined spaces including horse trailers, ditches, ravines, tree forks, mud, wells, and damaged or improper fencing. In addition, horses with extreme weakness following an injury or a fall, or with a history of neurologic or musculoskeletal diseases can be unable to stand, making transport and even survival difficult [1,2,3,4,5]. Tactical large animal rescue is a field that has evolved to aid animals in these situations. Because of the hazards of the size of the animals and the behavior of horses, it is critical that those attempting to work with recumbent or stranded animals have proper training, and immediate access to equipment to aid in the rescue process. Furthermore, due to the nature of equids as prey animals, the recumbent or stranded equid may struggle incessantly, often leading to severe musculoskeletal injuries and exhaustion [3]. Prolonged recumbency of large animals is associated with secondary injuries including cranial trauma, eye injuries, myopathies, and nerve paralysis [3,4,6].

Effective lifting devices used in large animals must utilize the skeletal system for support due to the considerable weight of these animals. While a number of devices have been developed in the last fifteen years to effectively aid vertical lifting of horses, many of them are complex systems and require frequent training of personnel; equipment is not readily available at the site of an incident, is cumbersome to move and store, and expensive [2,7,8,9,10,11]. Two of those devices, namely the Anderson Sling Support Device and the UC Davis Large Animal Lift, were developed by the first author [9,12]. The Anderson Sling Support Device was developed as a full body support sling for extended support and airlifting [9]. This device was later used as a recovery aid from general anesthesia in horses [10,11]. The UC Davis Large Animal Lift was developed to fulfill the need of a lightweight device that was easier to apply to the recumbent horse and for shorter use than the Anderson Sling Support Device [12]. The Animal Rescue and Transportation sling is a system used in Europe for both horses and cattle and provides general body support [7]. Complications associated with recumbency such as pressure sores, myopathies, neuropathies, and ileus can be prevented, and survival improved with the prompt use of these devices [3]. A more simple, fast to apply, inexpensive, more readily available at the incident, and easy short-term system to use in emergency situations is deemed necessary.

In this study, we report the use of a novel four round sling system referred to as the Loop Vertical Lift System or Loops System. The purpose of the study was to describe and evaluate the use of the Loop Vertical Lift System as a simple, quick option for vertical lift of equids.

## 2. Materials and Methods 

### 2.1. Loop Vertical Lift System on Live, Standing, Sedated Horses

All procedures involving live animals were approved by the Institutional Animal Care and Use Committee (IACUC) at the University of California, Davis. A pilot experiment consisting of a brief lift was undertaken prior to the full experiment. The pilot experiment was performed with one horse to assess sedation needs and general approach for Loop placement. The horse in the pilot experiment received 0.01 mg/kg of detomidine hydrochloride intravenously (IV), prior to placement of an intravenous catheter, then the loops were placed, and the horse was lifted 26 min after sedation administration. Upon initial suspension and the loops moving through the hind limbs, the horse began kicking with each hind limb, double-barrel kicking and bucking. This sedation protocol was deemed inadequate for that particular horse, and modifications were made to the sedation protocol. Additionally, we incorporated the use of foam cushions placed over the loop straps in the hind limb to prevent the straps from wedging between the hind limbs (shown in following sections). The purpose of the study was to describe and evaluate if the loops system when supporting the horse with non-weight bearing for a reasonable amount of time as expected for lifting a recumbent horse, would cause any harm, and be tolerated by the horse.

#### 2.1.1. Animals

Six geldings from the Center for Equine Health herd at the UC Davis School of Veterinary Medicine participated in this study. The animals were transported to the William R. Pritchard Veterinary Medical Teaching Hospital on the day of testing. The horses ranged in age from 6 to 15 years (mean of 9.7, SD ± 3.1 years), ranged in weight from 507 to 640 kg (mean of 578.7, SD ± 49.5 kg) and included three Thoroughbred, two Quarter Horses, and one Oldenberg.

#### 2.1.2. Physical Evaluation

The horses underwent a complete physical examination, and a brief lameness exam on the day prior to the lifting procedure. Immediately after the lifting procedure, a brief physical examination was performed, and one hour after the animals fully recovered from sedation their ability to ambulate at a walk was evaluated and classified as being normal or abnormal. At 24 h post-lifting procedure, the horses underwent another complete physical and brief lameness examination. The examinations were performed by two board certified equine medicine veterinarians.

Assessment of potential pressure points by the loops such as in the axillary, xiphoid, inguinal, and sheath areas for the presence of edema and pain was performed. Edema was classified on a scale of 0 (no edema), 1 (mild: minimally visible but detected upon palpation), 2 (moderate: visibly detected) to 3 (severe edema: visibly detected and pitting upon palpation). Pain was assessed using the Colorado Equine Comfort Assessment Scale, ranging from 0 to 4 (Colorado State University Veterinary Medical Center, 2007) [13]. To assess lameness, the horses were walked and briefly trotted in a straight line, then moved in a circle clockwise and anti-clockwise while being observed by the two veterinarians. Lameness was graded according to a simplified lameness scale because a full lameness exam was not performed. Briefly, lameness was classified as: 0 = if lameness was not perceived in any circumstances; 1 = if lameness was not consistently apparent in at (walk, trot or circles; 2 = if lameness was only consistently apparent at the circles, but not when trotting in a straight line, and 3 = if lameness was consistently apparent at a trot in a straight line; and 4 = if lameness was consistently apparent at a walk in a straight line. The purpose being to determine if adverse changes had occurred from the pressure of the loops system during the lift.

#### 2.1.3. Sedation and Catheter Protocol

Sedation was performed by IV administration of detomidine, at doses to achieve the desired effect and taking into consideration the individual horse’s demeanor, temperament and level of excitement due to environmental stimuli. Based on the findings of the pilot experiment, a two-step sedation protocol was instituted. The initial sedation was aimed to achieve the horse’s cooperation in order to place the intravenous catheter ensuring prompt venous access during the procedure, whereas the second sedation was aimed to have deeper sedation manifested by the horse’s head hanging low to the ground. Each horse received an initial dose of detomidine administered IV prior to placement of a Mila^®^ jugular catheter. In the initial sedation, each horse received either 3 or 5 mg of detomidine IV (0.005 to 0.009 mg/kg; Table 1), depending on the horses’ demeanor and level of excitement. Once the horse appeared sedated, the area over the jugular vein was clipped and aseptically prepared. A 14 gauge, 5 ¼ inch Mila^®^ catheter was placed in the left or right jugular vein and sutured into place using 2-0 nylon suture. The horses were walked to the padded anesthesia recovery stall, and the round slings were placed following a systematic procedure for placement of the Loop Vertical Lift System as described below. A second dose of detomidine was administered to each horse via the IV catheter (either 5, 7 or 10 mg of detomidine (0.008 to 0.018 mg/kg; Table 1), depending on the horse’s response to the first sedative and their level of excitement). The doses and the interval between sedation administration were recorded.

#### 2.1.4. Placement of the Loop Vertical Lift System on a Standing, Sedated Horse

The components of the Loop Vertical Lift System included four Lift-All EN60 × 6 FT Green Tuflex Polyester Round Loops [14], four Petzl^®^ OK screw lock carabiners, one ⅞ × 4 ½ inch bolt-type D-ring anchor shackle with a ¾ × 3 ¼ inch pin, and duct tape. Two people applied the sling to 3 horses and 3 people were used to apply the sling to the other 3 horses. Duct tape was used to maintain the round loops in place while setting up the shackle, as the duct tape was expected to break apart as the horse is lifted and the loops expanded. Each of the Lift-All Tuflex Green EN60 Roundslings has a rated capacity in pounds ranging depending on use from 4200 (choker), 5300 (vertical), 7400 (basket 45°), and 10,600 (basket 90°); and meet or exceed the Occupational Safety and Health Administration and American Society of Mechanical Engineers (OSHA, ASME) B30.9 standards and regulations (Lift-All 2014). The anchor or D-ring screw shackle had a rated capacity of 57,000 lbs (28.5 tons). The cost of this equipment was under $350.00 USD.

**Figure 1:**
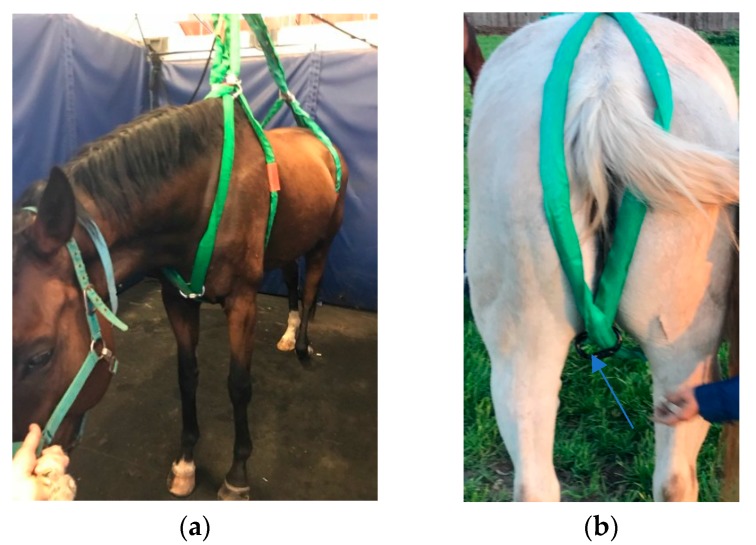

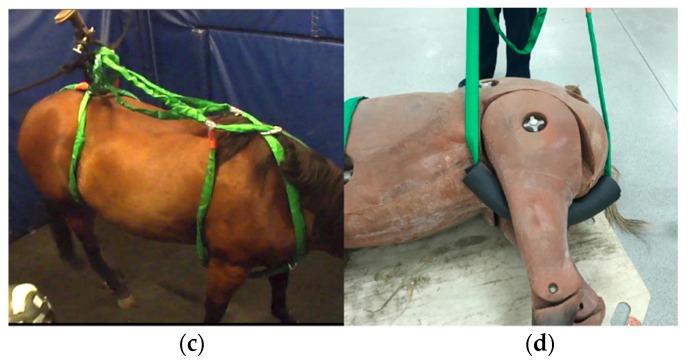
Placement of the Loop Vertical Lift System. (**a**). Placement of loops on the cranial aspect of the horse’s body. Carabiners are used to hold the loops in place at the sternum and withers. (**b**). Placement of loops on the caudal aspect of the horse’s body. (**c**). Full Loop Vertical Lift System attached to the anchor shackle and electric winch. (**d**) Foam cushion on the loop between the hind limbs. Duct tape is used to keep loops properly placed during application of the Loop Vertical Lift System.

Once the lightly sedated horse was in the padded anesthesia recovery stall, the Loops Vertical Lift System was placed following a systematic approach. Those persons working with the horses wore helmets and gloves. The first-round sling loop was placed around the left front limb by lifting the left front limb, then it was pulled up under the elbow and slipped over the horse’s head. Duct tape was placed around the two straps of the round loop above the withers and was used to hold the round sling loop in place against the body of the horse while the remaining loops were placed. The second-round sling loop was placed in the same manner on the right side of the horse so that the two round slings loops crossed each other at the sternum. The two separate round sling loops were clipped together both at the sternum and at the withers using two of the carabiners to keep the round loops from slipping in the girth area (Figure 1a). The third-round sling was placed around the left hind limb by lifting the limb, then it was pulled up over the limb, the tail was pulled through the round loop, and the two straps of the round sling were duct taped over the croup so that the straps were held in place against the body of the horse. The last round sling was placed in the same manner on the right hind limb of the horse, such that the round slings crossed each other underneath the tail and between the hind limbs. Carabiners were used to clip the round loops together both at the croup and under the tail (Figure 1b).

Once all four round slings were all held together on the back of the horse, the shackle was opened and each of the round loops was placed into the shackle in the center of the horse’s body. For safety, two ropes were tied from the shackle to metal rings in the back corners of the recovery stall in order to secure the shackle, and prevent the horse from lunging forward out of the stall.

Two lead ropes were also fed through the stall doors so individuals could control the head of the horse while outside of the stall. Finally, the anchor shackle was attached to the electric winch system via the latched rigging clip on the end of the chain (Figure 1c). Foam cushions were used over the loops in the hind limbs (Figure 1d).

#### 2.1.5. Lift Protocol Using the Loop Vertical Lift System

Once the Loop Vertical Lift System was in place, the horse was visually evaluated for level of sedation and given the second dose of detomidine as described earlier. Lifting was initiated 5 min after the second sedation administration.

Each of the six horses were lifted a few inches off the ground with an overhead chain hoist for 3 min within the closed-door recovery stall, and then lowered to the ground and allowed to stand. All of the entire lifting procedures were digitally recorded for later evaluation. At the end of three minutes of lifting, the horses were allowed time to stand unassisted for several minutes, then the loops were removed by letting them slide to the ground. Horses then were walked to adjacent recovery stalls. Once horses were willingly able to hold their head up (thirty minutes to one-hour post-sedation), the IV catheter was removed and the horses’ ability to ambulate at the walk was evaluated.

#### 2.1.6. Lift Evaluation

Subjective evaluation of lift quality as reported for other sling systems was performed using video recordings of the entire lift procedure for each of the six subjects while suspended and while being lowered. The lifts were scored as excellent, very good, good, or poor. If movement occurred while suspended, it was displayed by tossing/shaking of the head, twisting of the body, and moving of the limbs as if trying to gallop or buck. The length of the movement was recorded in seconds, and the time when movement started during the suspension was recorded in minutes and seconds. Once the horses’ feet touched the ground, the duration of any movement was recorded. Standing time was defined as the time between being lowered to the point of hooves touching the ground until able to fully support weight on all four limbs.

#### 2.1.7. Data Analysis

The data for each part of the study were summarized and reported using descriptive statistics. Data were displayed in tabular form whenever possible.

## 3. Results

### 3.1. Loop Vertical Lift System on Live, Standing, Sedated Horses

A summary of the sedation protocol for each horse is depicted in Table 1. The initial dose of detomidine ranged from 0.005 to 0.009 mg/kg, whereas the second dose of detomidine ranged from 0.008 to 0.018 mg/kg, with an average total dose of 0.02 mg/kg. The average time between the initial sedation and the second sedation administration was 24 min.

### 3.2. Loop Vertical Lift System Outcomes

All horses were lifted, suspended for 3 min and lowered successfully. Some brief movement/struggling behavior during the lifting procedure was displayed by four of the six horses, and in two horses during the lowering procedure (Table 2 and Table 3). None of the horses moved for longer than 15 s out of the 180 s lifting period. One horse (horse 2) was lowered for strap adjustment and then resuspended. In this horse, the slight movement observed during the first suspension and the marked movement during the lowering process were likely due to the strap positioning. Once the straps were adjusted, the horse remained quiet during suspension and during the lowering process. Transient movement during suspension occurred once in horses 3 and 6, and twice in horse 5; and in all cases the movement was followed by quieting down. One horse (horse 1) kicked once during the lowering process. Overall, lift behavior was considered excellent (n = 1/6), very good (n = 3/6) good (n = 2/6) and poor (n = 0/6). The first test horse which reacted poorly to the loops between its hind limbs was retested using foam tubes placed over the loop straps which created less pinch in the groin area (Figure 1d).

### 3.3. Evaluations before and after Lifting Procedure

No horses developed significant abnormal physical exam findings post lifting. Horses 1 through 4 had a prior history of lameness and/or musculoskeletal problems noted in their medical records. Physical examinations prior to the lifting procedure revealed no abnormalities in any of the 6 horses. None of the horses had any signs of pain or edema in the sheath, or in the axillary, xiphoid and inguinal areas before the lift. One horse (horse 2) was noted to have a mild, inconsistent lameness at the trot (Grade 1) before the lift.

All horses were able to ambulate normally at the walk thirty minutes to one hour after the lift. Physical examinations after the lifting procedure revealed moderate sheath edema in horse 3. The lameness in Horse 2 observed prior to the lift procedure was unchanged after the lifting procedure. The other subjects did not demonstrate any lameness before or after the lift.

### 3.4. Loop System Placement for Vertical Lift System Timing

The overall time for placement of the Loop Vertical Lift System for the two-person team ranged from 43 to 82 s (mean of 61 + 13 s), and for the three-person team ranged from 22 to 40 s (mean of 27 + 6 s).

## 4. Discussion

The present study describes a novel vertical lift procedure using a simple lift device to be used in large animal rescue for quick and safe vertical lift of stranded or recumbent horses, and may be suitable for mules or donkeys. The major advantages of this novel vertical lift are: (1) provides short-term full support using the skeletal system of the horse without causing injury to muscles, nerves or vasculature; (2) placement is fast and safe for personnel; (3) the system does not require J-hooks or knots; (4) it is a compact and portable device which can employ commercially available and reasonably priced equipment. The greater accessibility of this novel system at an incident may facilitate the rescue of stranded and recumbent equids, thus having a positive impact in animal welfare.

The two-step protocol of sedation provided an adequate chemical restraint for the study. The initial dose of detomidine hydrochloride at 0.005 to 0.009 mg/kg was adequate for the purpose of placing the intravenous catheter and the Loop System. The subsequent dose of detomidine appeared to provide sedation with less struggling when higher doses of 0.016 to 0.018 mg/kg were given. Furthermore, the two horses that received the higher doses of sedation were the ones that tolerated the procedure without any struggling. It is also possible that the horse’s demeanor and temperament influenced the level of sedation.

This study evaluated the set time for vertical non-weight bearing lifting of the horses as 3 min. The 3-min guideline used in this study was to simulate extreme conditions and should be the maximum time that a horse is allowed to hang non-weight bearing. On average, horses began some movement while suspended within 100 s of being lifted. When the horse first began movement, it was interpreted as the point at which the horse became uncomfortable. As a result of the observations in this study, it is of the authors’ opinion that the Loop Vertical Lift System is not designed for longer-term support, and is to be utilized for emergency lifting, rescue of a stranded or recumbent equid, or extraction from a confined space situation. Thus, it is not recommended to use the Loop Vertical Lift System for longer than the tested 3 min. Furthermore, studies should be conducted with a larger sample size, and with mules and donkeys to confirm our conclusion. It should be noted that in other studies using more extensive body support systems such as the UC Davis Anderson Sling, horses while non-weight bearing exhibited some degree of struggling and body movement as well [10,11].

While the study did not involve mules or donkeys, the similar anatomy for skeletal system support would suggest that this system may be appropriate for these equids. Different behavioral responses might occur with donkeys or mules and sedation protocols would have to be adjusted for that species.

Lastly, anyone working around a recumbent horse or attempting a vertical lift should be trained in safety measures for such a procedure before attempting the use of the Loops System. Safety equipment such as helmets, and gloves should be considered. These situations are termed low-frequency high-risk events and there is substantial risk of significant injury to humans attempting to aid a horse, donkey or mule that is in adverse circumstances. The use of this loops system for other rescue procedures other than vertical lift will be described in a companion paper [15] and will provide detailed step-by-step instructions.

## 5. Conclusions

The application of the Loop Vertical Lift System described in this paper provides a useful tool for first responders, veterinarians, and competent trained horse handlers/owners, thus greatly facilitating the success of large animal rescue. Lifting any equid should be done with safe and effective sedation performed by a veterinarian after assessment of the patient. This Loops System can be easily stored in a small duffle bag and is affordable compared to other devices such as the UC Davis Large Animal Lift. This is especially important for equipment that would be infrequently used but essential in an emergency situation. Anyone using this system should have formal animal rescue training for human and animal safety purposes. Future studies are recommended to evaluate the field use of the Loop Vertical Lift System in the rescue of stranded or recumbent equids.

## Figures and Tables

**Table 1:** Summary of sedation protocol. Doses of IV detomidine hydrochloride given at the initial sedation (for placement of the IV catheter) and at the second sedation (for lifting procedure) were chosen according to the level of excitement due to environmental stimuli and horse’s demeanor.

Horse Number	Initial Sedation Detomidine (mg/kg)	Second Sedation Detomidine (mg/kg)	Time between Doses (Min)	Total Sedative Detomidine (mg/kg)
1	0.005	0.018	24	0.023
2	0.009	0.009	26	0.018
3	0.008	0.008	28	0.016
4	0.008	0.016	24	0.024
5	0.006	0.014	18	0.020
6	0.005	0.013	21	0.018
Mean ± SD	0.007	0.013	24 ± 4	0.020

**Table 2:** Behavior observed during lifting with the Loop Vertical Lift System. The behavior observed is reported below—no motion, + 5–10 motions, ++ 10 or more motions. DL = double limb kick, SL = single limb.

	Movements While Suspended			Movements When Lowered		
Horse Number	Head Tossing	Body Twisting	Limbs Moving	Head Tossing	Kicking	Bucking
1	−	−	−	−	SL once	−
2	+	+	−	++	+	+
	−	−	−	−	−	−
3	+	++	+	−	−	−
4	−	−	−	−	−	−
5	+	++	+	++	+ DL	+
6	++	++	++	−	−	−

**Table 3:** Movement and time to stand during lifting with the Loop Vertical Lift System. Times are reported in seconds (s).

Horse	Time When Movement Occurred during Suspension	Length of Movement during Suspension	Length of Movement When Lowered	Time to Stand
1	No movement	No movement	No movement	5 s
2 *	at 40 s	2 s	10 s	12 s
	No movement	No movement	No movement	3 s
3	Once at 2 m 5 s	For 12 s	No movement	4 s
4	No movement	No movement	No movement	3 s
5	Twice: at 76 s; 100 s	Twice: for 7 s; 5 s	5 s	7 s
6	At 173 s	For 8 s	No movement	26 s

* Horse was lowered for strap adjustment and resuspended, thus movement during suspension and movement when lowered were recorded twice.
